# Supplementary material for: U.S. Fruit and Vegetable Affordability on the Thrifty Food Plan Depends on Purchasing Power and Safety Net Supports
Source: Int J Environ Res Public Health. 2022 Feb 27;19(5):2772. doi: 10.3390/ijerph19052772 (PMC8910477; doi:10.3390/ijerph19052772)
Supplement: Supplementary file 1 [file ijerph-19-02772-s001.zip › ijerph-1573407-supplementary.pdf]

```
new; cls;

filename = "u:\\CostFVSabrina\\Costs2020";
outfile ="u:\\CostFV4\\Savings.out";
save path= u:\CostFV4;
load path= u:\CostFVSabrina;

open input = ^filename VARINDXI;

print rowsf(input);

print input;

data = readr(input, ROWSF(input));

names = getnamef(input);

check1 = close(input);



n = rows(data);
itemcode = data[.,iitemcode];
juice = data[.,ijuice];
cost2016 =  data[.,icost2016];
cost =  data[.,icost2020];
groupcode =  data[.,iGROUPCOD];

/*****************************************************************************************
******************************************
```

```
*************************************************************************************
*****************************************

  Specify the number of combinations to create, the price thresholds for each bin, and insert the number
of

  half cup-equivalents from each group. However, you will generate some combinations in which fruit
juice accounts

  for more than half of all consumed fruit . In fact, as you create baskets with a greater share of items
from the

  first bin (cheapest price range), that share will grow.  You must therefore generate extra fruit
combinations

  beyond the number of combinations you want to simulate. You can throw away those illegimate
combinations.

  You specify the how many extra fruit combinations to generate using juicefactor = 2,3,4,... to create
twice, three

  times as many, etc. juice combinations.


  You cannot use simple random sampling. For example, weights = [3,1,0] will get you baskets with about
50% of items

  from bin 1, on average, though some may have as  few as 40% and others have as many 60% from bin
1.  Also, using

  weights = [7.5,1,0] will get you a basket with about 70% of items from bin 1. And using weights =
[31,1,0] will get

  you a basket with about 90% of items from bin 1.


*************************************************************************************
*****************************************

*************************************************************************************
*****************************************/


bss = 1000;

thresholdprice1 = 0.45;

thresholdprice2 = 0.90;
```

```
weightbin1 = 2.5;

weightbin2 = 1;

weightbin3 = 0;

juicefactor = 50;


/***********************************************************************************
*****************************************

************************************************************************************
*****************************************


  Specify the amount of foods the household must eat in half cup-equivalents, and their budget.


************************************************************************************
*****************************************

************************************************************************************
*****************************************/



numfruit = 104;

numdg = 14;

numrd = 46;

numlg = 14;

numstrch = 44;

numother = 36;


SNAP = 157;

FVbudget1 = SNAP;

FVbudget2 = 1.21*SNAP;
```

```
print "We generated";; print bss;; print "this many combinations of fruits and vegetables that would satsify DGAs.";


/************************************************************************************
*****************************************

*************************************************************************************
*****************************************

*********************************************** Generate the weights
*******************************************************

*************************************************************************************
*****************************************

*************************************************************************************
*****************************************/


binweight = zeros(n,1);

for c(1,n,1);

if cost[c,1] < thresholdprice1; binweight[c,1] = weightbin1;

elseif cost[c,1] < thresholdprice2; binweight[c,1] = weightbin2;

else; binweight[c,1] = weightbin3;

endif;

endfor;


x = groupcode~itemcode~juice~cost~binweight;


/************************************************************************************
*****************************************

*************************************************************************************
*****************************************

*********************************************** Generate fruit combinations
************************************************
```

```
**************************************************************************************************
***********************************************

**************************************************************************************************
***********************************************/



print "Each combination includes:";

print "Number of fruit half cup-equivalents (servings) is";; print numfruit;


fruits = selif(x, x[.,1] .== 1.0);

fruitcosts = fruits[.,4]./2;

nfruit = rows(fruits);


/* Picture an empirical density function.  For the nth fruit or vegetable in the group, the second column
gives the item number and the third column gives the left side of the bar on the number line. */


sumfruitweights = sumc(fruits[.,5]);

fruitweights = fruits[.,5]/sumfruitweights;


shares = zeros(nfruit+1,3);

shares[1,1] = 1;

shares[1,2] =fruits[1,2];

shares[1,3] = 0;


for i(2,nfruit,1);

shares[i,1] = i;

shares[i,2] = fruits[i,2];

shares[i,3] = shares[i-1,3] + fruitweights[i-1,.];

endfor;
```

```
shares[nfruit+1,1] = nfruit+1;

shares[nfruit+1,3] = 1;


/* Sample with replacement numfruit different fruits. Fruitbasket holds number of servings of each fruit
to include in the basket. */


bssfruit = juicefactor*bss;

fruitbasket = zeros(nfruit,bssfruit);


for b(1,bssfruit,1);


bb=rndu(numfruit,1);

bb = sortc(bb,1);



/*For each value of the uniform random sample that I generated, the following

"do loop" finds the appropriate location in the cumulative histogram.

I start by tallying the number of times the last fruit in my data is in the

combination.  This avoids the problem of the index being out of range. */


e1 = zeros(numfruit,1);

for c(1,numfruit,1);

if bb[c,1] < shares[2,3]; e1[c,1] = 1;

endif;

endfor;


first = sumc(e1);


e2 = zeros(numfruit,1);
```

```
for c(1,numfruit,1);
if bb[c,1] >= shares[nfruit,3]; e2[c,1] = 1;
endif;
endfor;

last = sumc(e2);

/* You delete rows that belong to the first and last PSU, if random number fell in first or last interval.
   Then you do the others, but code only works for those somewhere in the middle of the interval */

e = e1+e2;

bbb = delif(bb,e);
bk = rows(bbb);

others = zeros(nfruit,1);

if numfruit == first + last;
fruitbasket[.,b] = first|others[2:nfruit-1,1]|last;

else;

for c(1,bk,1);
r=2;
do until shares[r-1,3] < bbb[c,1] < shares[r,3]  ;
r=r+1;
endo;
k=r-2;
others[k,1] = others[k,1] + 1;
```

```
endfor;

fruitbasket[.,b] = first|others[2:nfruit-1,1]|last;

endif;

endfor;

juicechecker = zeros(nfruit,bssfruit);
for p(1,bssfruit,1);
juicechecker[.,p] = fruitbasket[.,p].*fruits[.,3];
endfor;

juicetest = sumc(juicechecker);
flag = zeros(bssfruit,1);
for j(1,bssfruit,1);
if juicetest[j,1] > 0.5*numfruit; flag[j,1] = 1;  endif;
endfor;

combos2 = flag~fruitbasket';

fruitbasket2 = selif(combos2, combos2[.,1] .== 0.0);
fruitbasket3 = fruitbasket2[.,2:nfruit+1]';
fruitbasket4 = fruitbasket3[.,1:bss];
```

```
/*********************************************************************************************
*****************************************

**********************************************************************************************
*****************************************

****************************************************   Generate dark green combinations
*****************************************************

**********************************************************************************************
*****************************************

**********************************************************************************************
*****************************************/


darkgreens = selif(x, x[.,1] .== 2.0);

dgcosts = darkgreens[.,4]./2;

ndg = rows(darkgreens);

print "Number of dark green vegetable half cup-equivalents (servings) is";; print numdg;


/* Picture an empirical density function.  For the nth fruit or vegetable in the group, the second column

gives the item number and the third column gives the left side of the bar on the number line. */


sumweights = sumc(darkgreens[.,5]);

weights = darkgreens[.,5]/sumweights;


shares = zeros(ndg+1,3);

shares[1,1] = 1;

shares[1,2] =darkgreens[1,2];

shares[1,3] = 0;


for i(2,ndg,1);

shares[i,1] = i;

shares[i,2] = darkgreens[i,2];
```

```
shares[i,3] = shares[i-1,3] + weights[i-1,.];

endfor;

shares[ndg+1,1] = ndg+1;

shares[ndg+1,3] = 1;


/* Sample with replacement numsize different fruits.  This may be number of full or half cup-
equivalents.  You will need to cut

cost in half at the end if you go with half cup-equivalents. */



dgbasket = zeros(ndg,bss);



for b(1,bss,1);


bb=rndu(numdg,1);

bb = sortc(bb,1);



/*For each value of the uniform random sample that I generated, the following

"do loop" finds the appropriate location in the cumulative histogram.

I start by tallying the number of times the first and last fruit in my data are in the

combination.  This avoids the problem of the index being out of range. */


e1 = zeros(numdg,1);

for c(1,numdg,1);

if bb[c,1] < shares[2,3]; e1[c,1] = 1;

endif;

endfor;
```

```
first = sumc(e1);

e2 = zeros(numdg,1);
for c(1,numdg,1);
if bb[c,1] >= shares[ndg,3]; e2[c,1] = 1;
endif;
endfor;

last = sumc(e2);

/* You delete rows that belong to the first and last PSU, if random number fell in first or last interval.
   Then you do the others, but code only works for those somewhere in the middle of the interval */

e = e1+e2;

bbb = delif(bb,e);
bk = rows(bbb);

others = zeros(ndg,1);

if numdg == first + last;
dgbasket[.,b] = first|others[2:ndg-1,1]|last;

else;

for c(1,bk,1);
r=2;
do until shares[r-1,3] < bbb[c,1] < shares[r,3]  ;
```

```
r=r+1;

endo;

k=r-2;

others[k,1] = others[k,1] + 1;

endfor;


dgbasket[.,b] = first|others[2:ndg-1,1]|last;


endif;


endfor;




/************************************************************************************
******************************************

*************************************************************************************
******************************************

*********************************************  Generate red and orange combinations
*********************************************

*************************************************************************************
******************************************

*************************************************************************************
******************************************/


redveggies = selif(x, x[.,1] .== 3.0);

redcosts = redveggies[.,4]./2;

nrd = rows(redveggies);

print "Number of red and orange vegetable half cup-equivalents (servings) is";; print numrd;
```

/* Picture an empirical density function. For the nth fruit or vegetable in the group, the second column gives the item number and the third column gives the left side of the bar on the number line. */

```
sumweights = sumc(redveggies[.,5]);

weights = redveggies[.,5]/sumweights;

shares = zeros(nrd+1,3);

shares[1,1] = 1;

shares[1,2] =redveggies[1,2];

shares[1,3] = 0;

for i(2,nrd,1);

shares[i,1] = i;

shares[i,2] = redveggies[i,2];

shares[i,3] = shares[i-1,3] + weights[i-1,.];

endfor;

shares[nrd+1,1] = nrd+1;

shares[nrd+1,3] = 1;
```

/* Sample with replacement numsize different fruits. This may be number of full or half cup-equivalents. You will need to cut

cost in half at the end if you go with half cup-equivalents. */

```
redbasket = zeros(nrd,bss);

for b(1,bss,1);

bb=rndu(numrd,1);

bb = sortc(bb,1);
```

```
/*For each value of the uniform random sample that I generated, the following
"do loop" finds the appropriate location in the cumulative histogram.
I start by tallying the number of times the last fruit in my data is in the
combination.  This avoids the problem of the index being out of range. */

e1 = zeros(numrd,1);
for c(1,numrd,1);
if bb[c,1] < shares[2,3]; e1[c,1] = 1;
endif;
endfor;

first = sumc(e1);

e2 = zeros(numrd,1);
for c(1,numrd,1);
if bb[c,1] >= shares[nrd,3]; e2[c,1] = 1;
endif;
endfor;

last = sumc(e2);

/* You delete rows that belong to the first and last PSU, if random number fell in first or last interval.
   Then you do the others, but code only works for those somewhere in the middle of the interval */

e = e1+e2;

bbb = delif(bb,e);
bk = rows(bbb);
```

```
others = zeros(nrd,1);

if numrd == first + last;
redbasket[.,b] = first|others[2:nrd-1,1]|last;

else;

for c(1,bk,1);
r=2;
do until shares[r-1,3] < bbb[c,1] < shares[r,3]  ;
r=r+1;
endo;
k=r-2;
others[k,1] = others[k,1] + 1;
endfor;

redbasket[.,b] = first|others[2:nrd-1,1]|last;

endif;

endfor;



/********************************************************************************************
*****************************************

*********************************************************************************************
*****************************************
```

```
***********************************************   Generate legumes combinations
************************************************

*********************************************************************************************
*****************************************

*********************************************************************************************
*****************************************/


legumes = selif(x, x[.,1] .== 6.0);

legcosts = legumes[.,4]./2;

nlg = rows(legumes);

print "Number of legumes half cup-equivalents (servings) is";; print numlg;


/* Picture an empirical density function.  For the nth fruit or vegetable in the group, the second column
gives the item number and the third column gives the left side of the bar on the number line. */


sumweights = sumc(legumes[.,5]);

weights = legumes[.,5]/sumweights;


shares = zeros(nlg+1,3);

shares[1,1] = 1;

shares[1,2] =legumes[1,2];

shares[1,3] = 0;


for i(2,nlg,1);

shares[i,1] = i;

shares[i,2] = legumes[i,2];

shares[i,3] = shares[i-1,3] + weights[i-1,.];

endfor;

shares[nlg+1,1] = nlg+1;

shares[nlg+1,3] = 1;
```

```
/* Sample with replacement numsize different fruits.  This may be number of full or half cup-
equivalents.  You will need to cut

cost in half at the end if you go with half cup-equivalents. */



legumesbasket = zeros(nlg,bss);


for b(1,bss,1);


bb=rndu(numlg,1);

bb = sortc(bb,1);


/*For each value of the uniform random sample that I generated, the following

"do loop" finds the appropriate location in the cumulative histogram.

I start by tallying the number of times the last fruit in my data is in the

combination.  This avoids the problem of the index being out of range. */


e1 = zeros(numlg,1);

for c(1,numlg,1);

if bb[c,1] < shares[2,3]; e1[c,1] = 1;

endif;

endfor;


first = sumc(e1);


e2 = zeros(numlg,1);

for c(1,numlg,1);

if bb[c,1] >= shares[nlg,3]; e2[c,1] = 1;
```

```
endif;
endfor;

last = sumc(e2);

/* You delete rows that belong to the first and last PSU, if random number fell in first or last interval.
   Then you do the others, but code only works for those somewhere in the middle of the interval */

e = e1+e2;

bbb = delif(bb,e);
bk = rows(bbb);

others = zeros(nlg,1);

if numlg == first + last;
legumesbasket[.,b] = first|others[2:nlg-1,1]|last;

else;

for c(1,bk,1);
r=2;
do until shares[r-1,3] < bbb[c,1] < shares[r,3]  ;
r=r+1;
endo;
k=r-2;
others[k,1] = others[k,1] + 1;
endfor;
```

```
legumesbasket[.,b] = first|others[2:nlg-1,1]|last;

endif;

endfor;



/***********************************************************************************
*****************************************

************************************************************************************
*****************************************

**********************************************   Generate starchy combinations
***********************************************

************************************************************************************
*****************************************

************************************************************************************
*****************************************/

starchies = selif(x, x[.,1] .== 4.0);
starchycosts = starchies[.,4]./2;
ns = rows(starchies);
print "Number of starchy vegetable half cup-equivalents (servings) is";; print numstrch;

/* Picture an empirical density function.  For the nth fruit or vegetable in the group, the second column
gives the item number and the third column gives the left side of the bar on the number line. */

sumweights = sumc(starchies[.,5]);
weights = starchies[.,5]/sumweights;
```

```
shares = zeros(ns+1,3);

shares[1,1] = 1;

shares[1,2] =starchies[1,2];

shares[1,3] = 0;


for i(2,ns,1);

shares[i,1] = i;

shares[i,2] = starchies[i,2];

shares[i,3] = shares[i-1,3] + weights[i-1,.];

endfor;

shares[ns+1,1] = ns+1;

shares[ns+1,3] = 1;


/* Sample with replacement numsize different fruits.  This may be number of full or half cup-
equivalents.  You will need to cut

cost in half at the end if you go with half cup-equivalents. */



starchybasket = zeros(ns,bss);



for b(1,bss,1);


bb=rndu(numstrch,1);

bb = sortc(bb,1);



/*For each value of the uniform random sample that I generated, the following

"do loop" finds the appropriate location in the cumulative histogram.
```

I start by tallying the number of times the last fruit in my data is in the combination. This avoids the problem of the index being out of range. */

```
e1 = zeros(numstrch,1);
for c(1,numstrch,1);
if bb[c,1] < shares[2,3]; e1[c,1] = 1;
endif;
endfor;

first = sumc(e1);

e2 = zeros(numstrch,1);
for c(1,numstrch,1);
if bb[c,1] >= shares[ns,3]; e2[c,1] = 1;
endif;
endfor;

last = sumc(e2);

/* You delete rows that belong to the first and last PSU, if random number fell in first or last interval.
   Then you do the others, but code only works for those somewhere in the middle of the interval */

e = e1+e2;

bbb = delif(bb,e);
bk = rows(bbb);

others = zeros(ns,1);
```

```
if numstrch == first + last;

starchybasket[.,b] = first|others[2:ns-1,1]|last;


else;


for c(1,bk,1);

r=2;

do until shares[r-1,3] < bbb[c,1] < shares[r,3]  ;

r=r+1;

endo;

k=r-2;

others[k,1] = others[k,1] + 1;

endfor;


starchybasket[.,b] = first|others[2:ns-1,1]|last;


endif;


endfor;




/************************************************************************************
*****************************************

************************************************************************************
*****************************************

***********************************************  Generate other veggie combinations
************************************************

************************************************************************************
*****************************************
```

```
************************************************************************************
***************************************/

otherveggies = selif(x, x[.,1] .== 5.0);

othervegcosts = otherveggies[.,4]./2;

nov = rows(otherveggies);

print "Number of other vegetable half cup-equivalents (servings) is";; print numother;

/* Picture an empirical density function.  For the nth fruit or vegetable in the group, the second column gives the item number and the third column gives the left side of the bar on the number line. */

sumweights = sumc(otherveggies[.,5]);

weights = otherveggies[.,5]/sumweights;

shares = zeros(nov+1,3);

shares[1,1] = 1;

shares[1,2] =otherveggies[1,2];

shares[1,3] = 0;

for i(2,nov,1);

shares[i,1] = i;

shares[i,2] = otherveggies[i,2];

shares[i,3] = shares[i-1,3] + weights[i-1,.];

endfor;

shares[nov+1,1] = nov+1;

shares[nov+1,3] = 1;

/* Sample with replacement numsize different fruits.  This may be number of full or half cup-
equivalents.  You will need to cut
```

```
cost in half at the end if you go with half cup-equivalents. */


otherbasket = zeros(nov,bss);


for b(1,bss,1);

bb=rndu(numother,1);
bb = sortc(bb,1);


/*For each value of the uniform random sample that I generated, the following
"do loop" finds the appropriate location in the cumulative histogram.
I start by tallying the number of times the last fruit in my data is in the
combination.  This avoids the problem of the index being out of range. */

e1 = zeros(numother,1);
for c(1,numother,1);
if bb[c,1] < shares[2,3]; e1[c,1] = 1;
endif;
endfor;

first = sumc(e1);

e2 = zeros(numother,1);
for c(1,numother,1);
if bb[c,1] >= shares[nov,3]; e2[c,1] = 1;
endif;
```

```
endfor;

last = sumc(e2);

/* You delete rows that belong to the first and last PSU, if random number fell in first or last interval.
   Then you do the others, but code only works for those somewhere in the middle of the interval */

e = e1+e2;

bbb = delif(bb,e);
bk = rows(bbb);

others = zeros(nov,1);

if numother == first + last;
otherbasket[.,b] = first|others[2:nov-1,1]|last;

else;

for c(1,bk,1);
r=2;
do until shares[r-1,3] < bbb[c,1] < shares[r,3]  ;
r=r+1;
endo;
k=r-2;
others[k,1] = others[k,1] + 1;
endfor;

otherbasket[.,b] = first|others[2:nov-1,1]|last;
```

```
endif;

endfor;





/*********************************************************************************
****************************************

*********************************************************************************
****************************************

*********** Stack the subgroup combinations.  And check the number of fruits and veggies included
from each cost range  ***********************

*********************************************************************************
****************************************

*********************************************************************************
****************************************/


Combinations = fruitbasket4|dgbasket|redbasket|starchybasket|otherbasket|legumesbasket;

foodcosts = fruitcosts|dgcosts|redcosts|starchycosts|othervegcosts|legcosts;


bin1calculator = zeros(rows(Combinations),bss);

for p(1,bss,1);

for v(1,rows(combinations),1);

if foodcosts[v,.] < thresholdprice1/2; bin1calculator[v,p] = Combinations[v,p]; endif;

endfor;

endfor;
```

```
bin1total = sumc(bin1calculator);

bin1share = bin1total/(numfruit + numdg + numrd + numlg + numstrch + numother);


print "Share of baskets from bin 1 is";; print meanc(bin1share);




/***********************************************************************************
****************************************

************************************************************************************
****************************************

***********************************  Count the number of different types of products included.
*****************************************

************************************************************************************
****************************************

************************************************************************************
****************************************/


numcounter = zeros(rows(Combinations),bss);


for p(1,bss,1);


for c(1,rows(Combinations),1);

if Combinations[c,p] >= 1; numcounter[c,p] = 1; else; numcounter[c,p] = 0;  endif;

endfor;


endfor;


counters = sumc(numcounter);

counters2 = (sumc(counters))/bss;

print "The average number of different products in each basket is";; print counters2;
```

```
/**********************************************************************************
****************************************

***********************************************************************************
****************************************

************************   Check basket costs and required budget shares in scenario #1 .
******************************************

***********************************************************************************
****************************************

***********************************************************************************
****************************************/


foodcosts1 = foodcosts.*0.7;


pricechecker = zeros(rows(Combinations),bss);

for p(1,bss,1);

pricechecker[.,p] = Combinations[.,p].*foodcosts1;

endfor;



totalcost = (sumc(pricechecker));

pricetest = zeros(bss,4);

for p(1,bss,1);

pricetest[p,1] = p;

pricetest[p,2] = totalcost[p,.];

pricetest[p,3] = totalcost[p,.] / FVbudget1;

pricetest[p,4] = totalcost[p,.] / FVbudget2;

endfor;
```

```
print;

print "The average cost of the baskets at 70% of national average prices is";; print meanc(totalcost);

print;

print "Average required budget share with 100% of SNAP benefits is ";; print meanc(pricetest[.,3] );;

print "with a standard deviiation of ";; print stdc(pricetest[.,3] );;

print ", a minimum of ";; print minc(pricetest[.,3] );;

print ", and a maximum of ";; print maxc(pricetest[.,3] );;

print;

print;

print "Average required budget share with 121% of SNAP benefits is ";; print meanc(pricetest[.,4] );;

print "with a standard deviiation of ";; print stdc(pricetest[.,4] );;

print ", a minimum of ";; print minc(pricetest[.,4] );;

print ", and a maximum of ";; print maxc(pricetest[.,4] );;

print;

print;




/*********************************************************************************
****************************************

**********************************************************************************
****************************************

************************   Check basket costs and required budget shares in scenario #2 .
******************************************

**********************************************************************************
****************************************

**********************************************************************************
****************************************/
```

```
foodcosts1 = foodcosts.*0.85;


pricechecker = zeros(rows(Combinations),bss);
for p(1,bss,1);
pricechecker[.,p] = Combinations[.,p].*foodcosts1;
endfor;



totalcost = (sumc(pricechecker));
pricetest = zeros(bss,4);
for p(1,bss,1);
pricetest[p,1] = p;
pricetest[p,2] = totalcost[p,.];
pricetest[p,3] = totalcost[p,.] / FVbudget1;
pricetest[p,4] = totalcost[p,.] / FVbudget2;
endfor;


print;
print "The average cost of the baskets at 85% of national average prices is";; print meanc(totalcost);
print;
print "Average required budget share with 100% of SNAP benefits is ";; print meanc(pricetest[.,3] );;
print "with a standard deviiation of ";; print stdc(pricetest[.,3] );;
print ", a minimum of ";; print minc(pricetest[.,3] );;
print ", and a maximum of ";; print maxc(pricetest[.,3] );;
print;
print;
print "Average required budget share with 121% of SNAP benefits is ";; print meanc(pricetest[.,4] );;
print "with a standard deviiation of ";; print stdc(pricetest[.,4] );;
print ", a minimum of ";; print minc(pricetest[.,4] );;
```

```
print ", and a maximum of ";; print maxc(pricetest[.,4] );;

print;

print;



/**********************************************************************************
****************************************

***********************************************************************************
****************************************

************************    Check basket costs and required budget shares in scenario #3 .
******************************************

***********************************************************************************
****************************************

***********************************************************************************
****************************************/


foodcosts1 = foodcosts.*1.0;


pricechecker = zeros(rows(Combinations),bss);

for p(1,bss,1);

pricechecker[.,p] = Combinations[.,p].*foodcosts1;

endfor;




totalcost = (sumc(pricechecker));

pricetest = zeros(bss,4);

for p(1,bss,1);

pricetest[p,1] = p;

pricetest[p,2] = totalcost[p,.];

pricetest[p,3] = totalcost[p,.] / FVbudget1;
```

```
pricetest[p,4] = totalcost[p,.] / FVbudget2;

endfor;


print;

print "The average cost of the baskets at 100% of national average prices is";; print meanc(totalcost);

print;

print "Average required budget share with 100% of SNAP benefits is ";; print meanc(pricetest[.,3] );;

print "with a standard deviiation of ";; print stdc(pricetest[.,3] );;

print ", a minimum of ";; print minc(pricetest[.,3] );;

print ", and a maximum of ";; print maxc(pricetest[.,3] );;

print;

print;

print "Average required budget share with 121% of SNAP benefits is ";; print meanc(pricetest[.,4] );;

print "with a standard deviiation of ";; print stdc(pricetest[.,4] );;

print ", a minimum of ";; print minc(pricetest[.,4] );;

print ", and a maximum of ";; print maxc(pricetest[.,4] );;

print;

print;




/**********************************************************************************
****************************************

***********************************************************************************
****************************************

************************* Check basket costs and required budget shares in scenario #4 .
******************************************

***********************************************************************************
****************************************
```

```
*****************************************************************************************
*****************************************/

foodcosts1 = foodcosts.*1.15;

pricechecker = zeros(rows(Combinations),bss);
for p(1,bss,1);
pricechecker[.,p] = Combinations[.,p].*foodcosts1;
endfor;



totalcost = (sumc(pricechecker));
pricetest = zeros(bss,4);
for p(1,bss,1);
pricetest[p,1] = p;
pricetest[p,2] = totalcost[p,.];
pricetest[p,3] = totalcost[p,.] / FVbudget1;
pricetest[p,4] = totalcost[p,.] / FVbudget2;
endfor;

print;
print "The average cost of the baskets at 115% of national average prices is";; print meanc(totalcost);
print;
print "Average required budget share with 100% of SNAP benefits is ";; print meanc(pricetest[.,3] );;
print "with a standard deviiation of ";; print stdc(pricetest[.,3] );;
print ", a minimum of ";; print minc(pricetest[.,3] );;
print ", and a maximum of ";; print maxc(pricetest[.,3] );;
print;
print;
```

```
print "Average required budget share with 121% of SNAP benefits is ";; print meanc(pricetest[.,4] );;

print "with a standard deviiation of ";; print stdc(pricetest[.,4] );;

print ", a minimum of ";; print minc(pricetest[.,4] );;

print ", and a maximum of ";; print maxc(pricetest[.,4] );;

print;

print;



/***********************************************************************************
*****************************************

***********************************************************************************
*****************************************

*************************   Check basket costs and required budget shares in scenario #5 .
*******************************************

***********************************************************************************
*****************************************

***********************************************************************************
*****************************************/


foodcosts1 = foodcosts.*1.29;


pricechecker = zeros(rows(Combinations),bss);

for p(1,bss,1);

pricechecker[.,p] = Combinations[.,p].*foodcosts1;

endfor;



totalcost = (sumc(pricechecker));

pricetest = zeros(bss,4);

for p(1,bss,1);
```

```
pricetest[p,1] = p;
pricetest[p,2] = totalcost[p,.];
pricetest[p,3] = totalcost[p,.] / FVbudget1;
pricetest[p,4] = totalcost[p,.] / FVbudget2;
endfor;


print;
print "The average cost of the baskets at 129% of national average prices is";; print meanc(totalcost);
print;
print "Average required budget share with 100% of SNAP benefits is ";; print meanc(pricetest[.,3] );;
print "with a standard deviiation of ";; print stdc(pricetest[.,3] );;
print ", a minimum of ";; print minc(pricetest[.,3] );;
print ", and a maximum of ";; print maxc(pricetest[.,3] );;
print;
print;
print "Average required budget share with 121% of SNAP benefits is ";; print meanc(pricetest[.,4] );;
print "with a standard deviiation of ";; print stdc(pricetest[.,4] );;
print ", a minimum of ";; print minc(pricetest[.,4] );;
print ", and a maximum of ";; print maxc(pricetest[.,4] );;
print;
print;
```
